# Association between serum PGE_2_ levels and degree of acid-fast bacilli positivity in sputum of pulmonary tuberculosis patients

**DOI:** 10.1016/j.amsu.2021.103008

**Published:** 2021-11-03

**Authors:** Herley Windo Setiawan, Resti Yudhawati, Irmi Syafaah

**Affiliations:** a Department of Pulmonology and Respiratory Medicine, Faculty of Medicine, Universitas Airlangga – Dr. Soetomo General Academic Hospital, Surabaya, Indonesia; b Department of Pulmonology and Respiratory Medicine, Faculty of Medicine, Universitas Airlangga – Universitas Airlangga Teaching Hospital, Surabaya, Indonesia

**Keywords:** Positivity of acid-fast bacilli, Pulmonary tuberculosis, Serum PGE_2_ levels

## Abstract

**Background:**

*Mycobacterium tuberculosis* that infected apoptotic macrophages is triggered by PGE_2_. Apoptosis suppresses the growth of *Mycobacterium tuberculosis* bacteria, which is shown in the results of acid-fast bacilli (AFB) in the sputum that becomes a marker of the number of bacteria.

**Objective:**

Analyzing the association between serum PGE_2_ levels and the positivity of AFB in the sputum of tuberculosis patients.

**Methods:**

A cross-sectional study was carried out from August 2019–July 2020. Serum PGE_2_ levels and AFB levels in sputum were collected from participants. Data analysis used the Chi-square test and Spearman's correlation with *p* < 0.05.

**Results:**

The average participants’ serum PGE_2_ levels were 446.37 ± 510.27 pg/ml, with a median value of 216.95 pg/ml. Most participants had normal serum PGE_2_ levels (62.9%). Most participants had a high positivity of AFB in sputum (58.1%). Analysis of the association between serum PGE_2_ levels and the degree of AFB positivity in sputum obtained *r* = −0.036 and *p*-value = 0.780.

**Conclusion:**

There is a weak negative association between serum PGE_2_ levels and the degree of AFB positivity in sputum but not statistically significant.

## Highlights

• Serum PGE_2_ levels of tuberculosis patients tend to be normal even though acid-fast bacilli (AFB) values are high.
• Most of the new and recurrent cases of pulmonary tuberculosis patients had normal PGE_2_ levels.
• Serum PGE_2_ levels have a negative association with AFB value.

## 1 Introduction

Tuberculosis (TB) is still a global health problem [1]. The increase in TB cases is accompanied by an increase in drug-resistant TB (DR TB) cases. In the Global Tuberculosis Report, WHO reported that 10 million people were suffering from TB, both new and relapsed cases, with 558,000 of whom had DR TB [2]. Indonesia ranks third in the country with the highest TB incidence globally, both new and relapse cases. The number of new and relapsed TB cases in Indonesia in 2017 was 442,172, and 54% of them were confirmed bacteriologically by either acid-fast bacilli (AFB) sputum staining or sputum culture [3].

The pathogenesis of TB is an interaction between *Mycobacterium tuberculosis* and the host [4]. The process begins with alveolar macrophages and dendritic cells as the first cells facing *Mycobacterium tuberculosis* bacteria. Macrophages’ response as the mainline in dealing with *Mycobacterium tuberculosis* infection is influenced by various inflammatory mediators [5]. The failure of macrophages to control the number of *Mycobacterium tuberculosis* will result in the significant growth of bacteria [6,7].

This condition emphasizes the important role of the host immune system in determining the susceptibility of TB to relapse. Several studies pointed out that Prostaglandin E_2_ (PGE_2_) affects macrophages as the main cells in the innate immune system. PGE_2_ induces apoptosis and inhibits necrosis of macrophages infected with *Mycobacterium tuberculosis* [5,8,9]. Macrophage apoptosis is reported to reduce the growth rate of *Mycobacterium tuberculosis,* which is very important in the elimination mechanism of bacteria that infects the lungs, whereas necrosis plays the opposite role [5,8,10]. When the growth of *Mycobacterium tuberculosis* cannot be inhibited, the number of bacteria will increase. The high number of bacterias is reflected in the degree of phlegm AFB positivity. The higher the value of positivity for AFB in sputum, the greater the number of *Mycobacterium tuberculosis* bacteria contained in each ml of sputum [11]. The higher the number of bacterias, the easier it is can transmit, broader lung damage, and an increased risk of resistance [12,13].

Based on the facts above, this study further revealed the association between PGE_2_, which represents the innate immune system, and the degree of phlegm AFB positivity, which represents the number of bacterias. This research is important because no similar study was conducted in humans, so it is hoped that this research could provide further research.

## 2 Methods

### 2.1 Participants

Participants in this study were both new and relapsed patients with pulmonary tuberculosis. The inclusion criteria were patients diagnosed with pulmonary tuberculosis [3,14], positive sputum examination results for AFB, aged 21–65 years, who cooperated during the research procedure. Meanwhile, the exclusion criteria included patients with risk factors for immunocompromised (AIDS, malignancy, and systemic lupus erythematosus), patients having received anti-tuberculosis drug therapy for their current illness, patients taking non-steroidal anti-inflammatory drugs and/or corticosteroids in the past week.

### 2.2 Ethical clearance

Participants and their families filled out the consent form before the study. Participants filled out the consent form consciously and without coercion. This study received ethical approval based on the Declaration of Helsinki and obtained the registry of research at the Health Research Ethics Committee in the Hospital.

### 2.3 Study design

**Fig. 1:**
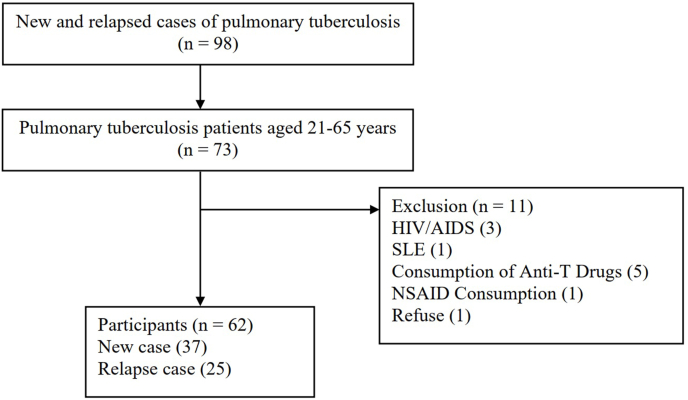
Participant requitement process.

A cross-sectional study was carried out from August 2019–July 2020. The number of participants in this study was 62 patients that were obtained using Ronald Fisher's classic z transformation formula. The sample collection used a consecutive sampling technique (Fig. 1). Serum PGE_2_ levels and levels of AFB in sputum were taken from the participants. This study report is by the Strengthening the Reporting of Cohort Studies in Surgery (STROCSS) 2019 guideline [15].

### 2.4 Measurement of serum PGE_2_ level

Serum PGE_2_ level is the total concentration of PGE_2_ in the blood of pulmonary tuberculosis patients. This examination was carried out by taking 3–5 ml of the patient's venous blood and analyzed using the Elisa Kit PGE_2_ (pg/ml). Serum PGE_2_ level is categorized into high if the value is more than 400 pg/ml, normal if the value is 200–400 pg/ml, and low if the value is less than 200 pg/ml [16].

### 2.5 Acid-fast bacilli test

Sputum examination was conducted to determine the degree of the participant's AFB positivity. Sputum collection for participants is carried out by the patient independently in the morning [17] which the participant gets an explanation form a pulmonary specialist regarding effective deep breathing and coughing techniques [18]. The sputum is put into a tube that has been prepared previously and then taken to the laboratory for analysis. The examination of AFB in the participant's sputum used the acid-fast staining method (Ziehl Nielssen) or the rapid molecular test of sputum with the GeneXpert machine [19]. The degree of phlegm AFB positivity was assessed based on the International Union Against Tuberculosis Lung Disease (IUATLD) standards which were categorized into 2: low (1+ and scanty) and high (2+ and 3+) [19,20].

### 2.6 Statistical analysis

The analysis in this study used descriptive analysis and bivariate analysis. Descriptive analysis included the presentation of the results descriptively using the distribution table, mean, median, standard deviation, maximum value, and minimum value. Meanwhile, bivariate analysis was used to assess the association between two variables. The association between variables was analyzed using the Chi-Square test and assessed the association strength using the Spearman correlation test. The analysis was declared significant if *p* < 0.05. The analysis was assisted by IBM SPSS Statistics software version 21.0 (IBM Corp., Armonk, NY, USA).

## 3 Results

### 3.1 Characteristic of participant

Most participants were male who was 43.37 ± 12.58 years old. Meanwhile, the median of participants’ age was 44.5 years, with the lowest age being 21 years and the highest being 64 years. Some patients had a smoking habit (56.5%) and comorbidity of diabetes mellitus (32.3%). A total of 37 participants were new tuberculosis patients and the rest were relapsed, tuberculosis patients. Most participants had a body mass index (BMI) in the skinny category as much as 53.2% (Table 1). The average BMI value was 19.46 ± 4.05 kg/m^2^, with a value range of 14.20–38.28 kg/m^2^.

**Table 1:** Characteristic of participant.

Variable	n (%)
Sex	
Male	36 (58.1)
Female	26 (41.9)
Education	
Elementary School	8 (12.9)
Junior High School	12 (19.4)
Senior High School	34 (54.8)
College	7 (11.3)
Not attending school	1 (1.6)
History of Diabetes Mellitus	
Yes	20 (32.3)
No	42 (67.7)
History of Tuberculosis Treatment	
New case	37 (59.7)
Relapse	25 (40.3)
Smoking Habit	
Smoking	35 (56.5)
No smoking	27 (43.5)
Degree of Acid-Fast Bacilli Positivity	
Low	26 (41.9)
High	36 (58.1)
Serum PGE_2_ Level	
Low	9 (14.5)
Normal	39 (62.9)
High	14 (22.6)
Body Mass Index	
Skinny (<18.5 kg/m^2^)	33 (53.2)
Normal (18.5–25.0 kg/m^2^)	24 (38.7)
Fat (>25.0 kg/m^2^)	5 (8.1)

### 3.2 Distribution of serum PGE_2_ levels in tuberculosis patients

Most participants had normal serum PGE_2_ levels (62.9%; Table 1). The average participants had serum PGE_2_ levels of 446.37 ± 510.27 pg/ml, with a median value of 216.95 pg/ml. The lowest and highest value of the participants’ serum PGE_2_ levels were 191.00 pg/ml and 2374.00 pg/ml, respectively. The serum PGE_2_ levels of smoking and non-smoking participants was 228.80 (191.0–2,3374.0) pg/ml and 214.40 (198.3–1724.0) pg/ml, respectively. Most serum PGE_2_ levels of smoking participants were normal (50%), while the serum PGE_2_ levels of non-smoking participants were mostly normal (78%; p = 0.053). The median value of serum PGE_2_ levels for participants with and without diabetes mellitus was 217.30 (191.0–1986.0) pg/ml and 216.80 (193.0–2374.0) pg/ml, respectively. The value of serum PGE_2_ levels of participants with and without diabetes mellitus were 45% and 71%, respectively, indicating that most participants had normal values (*p* = 0.118; Table 2).

**Table 2:** Distribution of serum PGE_2_ levels in tuberculosis patients.

Variable	Serum PGE_2_ Levels			*p*
	Low	Normal	High	
Pulmonary Tuberculosis				
New case	6 (16)	23 (62)	8 (22)	0.292
Relapse case	3 (12)	16 (64)	6 (24)	
Diabetes mellitus				
Yes	4 (20)	9 (45)	7 (35)	0.118
No	5 (12)	30 (71)	7 (17)	
BMI				
Skinny	1 (3)	24 (73)	8 (24)	0.058
Normal	7 (29)	12 (50)	5 (21)	
Fat	1 (20)	3 (60)	1 (20)	
Smoking				
Yes	6 (18)	17 (50)	11 (32)	0.053
No	3 (11)	22 (78)	3 (11)	

Abbreviation: BMI = body mass index.

Most of the participants’ serum PGE_2_ levels were normal in both groups of participants with a new diagnosis of pulmonary tuberculosis (62%) and relapsed (64%; *p* = 0.292). The median value of serum PGE_2_ levels for participants diagnosed with new pulmonary tuberculosis was 215.70 (191.0–1724.0) pg/ml and participants diagnosed with relapsed pulmonary tuberculosis was 224.40 (193.2–2374.0) pg/ml. Participants' serum PGE_2_ levels that were categorized by BMI were mostly normal, with 73% of skinny participants, 50% of normal participants, and 60% of fat participants (*p* = 0.058; Table 3). The median value of serum PGE_2_ levels of participants with BMI in the skinny category was 222.60 (194.3–1986.0) pg/ml, normal was 210.30 (191.0–2374.0) pg/ml, and fatwas 216.40 (199.0–1497.0) pg/ml.

**Table 3:** Distribution of positivity of acid-fast bacilli in sputum of tuberculosis patients.

Variable	Degree of Acid-Fast Bacilli Positivity		*p*
	Low (%)	High	
Pulmonary Tuberculosis			
New case	12 (32)	25 (68)	0.065
Relapse case	14 (56)	11 (44)	
Diabetes Mellitus			
Yes	7 (35)	13 (65)	0.455
No	19 (45)	23 (55)	
BMI			
Skinny	15 (45)	18 (55)	0.561
Normal	10 (42)	14 (58)	
Fat	1 (20)	4 (80)	
Smoking			
Yes	15 (45)	19 (56)	0.798
No	11 (39)	17 (61)	

Abbreviation: BMI = body mass index.

### 3.3 Distribution of positivity of acid-fast bacilli in sputum of tuberculosis patients

Most participants had a high degree of AFB positivity in sputum as much as 58.1% (Table 1). Most participants who were diagnosed with new cases of pulmonary tuberculosis had a high degree of AFB positivity (68%). Meanwhile, most participants diagnosed with relapsed pulmonary tuberculosis had a low positivity degree (56%; *p* = 0.065). Some participants had a high degree of AFB positivity in participants with and without a history of diabetes mellitus of 65% and 55%, respectively (*p* = 0.455). Participants' BMI was categorized into 3, namely skinny, normal, and fast, in which some participants had a high degree of AFB positivity (*p* = 0.561). Most smoking (56%) and non-smoking (61%) participants had high positivity of AFB (*p* = 0.798; Table 3).

## 4 Association between serum PGE_2_ levels and positivity of acid-fast bacilli in sputum of tuberculosis patients

The results showed that most participants with low (89%) and high (71%) serum PGE_2_ levels had a high positivity of AFB in sputum as much as 89%. Meanwhile, participants with normal serum PGE_2_ levels had a low positivity degree of AFB in sputum as much as 54% (*p* = 0.036). The strength of the association between serum PGE_2_ levels and the degree of AFB positivity in sputum obtained *r* = −0.036 and *p*-value = 0.780 (Table 4).

**Table 4:** Association between PGE_2_ levels and positivity of acid-fast bacilli in the sputum of tuberculosis patients.

Variable	Tuberculosis Positivity		*p*^a^	*r*	*p*^b^
	Low	High			
PGE_2_ Levels					
Low	1 (11)	8 (89)	0.036	−0.036	0.780
Normal	21 (54)	18 (46)			
High	4 (29)	10 (71)			

Note: *p*^a^ = Chi-square test; *p*^b^ = Spearman's correlation test.

## 5 Discussion

PGE_2_ is a derivative of arachidonic acid produced by various inflammatory cells, especially macrophages. PGE_2_, as an inflammatory mediator, plays a role in regulating various cell functions, namely macrophages, T cells, etc. In addition, PGE_2_ plays a role in various body functions such as blood pressure regulation, temperature regulation, gastric protection, and childbirth [21]. Under various conditions such as changes in environmental temperature, hunger conditions, stress, PGE_2_ will be produced so that levels in the body will rise and fall in various ways [22].

Schoenberger et al. reported an increase in serum PGE_2_ levels in patients with diabetic retinopathy [23]. A study conducted by Lo et al. showed that the increase in serum PGE_2_ levels was due to the upregulation of the cyclooxygenase-2 (COX_2_) enzyme in patients with diabetes mellitus [24]. Kumar et al. reported differences in plasma PGE_2_ levels in TB patients compared to TB-DM [16]. These results are inconsistent with various studies that reported increased levels of PGE_2_ in smokers. Amadio et al. reported an increase in PGE_2_ production in smokers due to the modulation of expression of tissue factors exposed to cigarette smoke [25]. Chen et al. in their study also reported the role of cigarette smoke in increasing PGE_2_ production [26].

The condition obtained in this study seemed to occur because of the patient's experience factor. In patients with relapse cases, the experience of suffering from TB in the past will make the patient who has a cough immediately come to the health facility. Meanwhile, new case patients ignore the cough complaint that leads to accompanying complaints such as weight loss, hemoptysis, or fever. When these accompanying complaints occur, the course of TB disease would be long enough to increase the number of bacterias [1].

The profile of serum PGE_2_ levels showed that the average participants had 446.23 pg/ml, with a standard deviation of 510.27 pg/ml. According to some literature, normal serum PGE_2_ levels range from 200 to 400 pg/ml [16]. PGE_2_ is a derivative of arachidonic acid produced mainly by inflammatory cells to face invading pathogens from outside. The effect of PGE_2_ will trigger apoptosis of macrophages infected with *Mycobacterium tuberculosis* [4]. Macrophage apoptosis will have an elimination effect because *Mycobacterium tuberculosis* bacteria can be destroyed. PGE_2_ also suppresses macrophage necrosis which can lead to bacterial dissemination. Increased levels of PGE_2_ are associated with a decrease in the number of bacteria in the lung [7].

The negative association between serum PGE_2_ levels and the degree of phlegm AFB positivity is by a study conducted by Dietzold and Amaral. Dietzold et al. reported that high levels of PGE_2_ and low levels of LXA_4_ suppress the growth of *Mycobacterium tuberculosis* [7]. Amaral et al. also reported that PGE_2_ is associated with macrophage apoptosis in vitro. Apoptotic macrophages infected with *Mycobacterium tuberculosis* will increase the elimination of these bacterias [4]. The two studies above reported a significant association between PGE_2_ and the growth of *Mycobacterium tuberculosis*. The statistical analysis results of this study showed that the association between serum PGE_2_ levels and the degree of AFB positivity was not statistically significant. The main difference between this study and the two studies above is that both were carried out on mice and in vitro, whereas this study was conducted on pulmonary TB patients with various complications and uncontrollable comorbidities.

The results of this study can be used as consideration for conducting further research on the predictor factors for the positivity of AFB in pulmonary TB patients. The use of PGE_2_ together with LXA_4_ is expected to be able to assist clinicians in predicting the level of AFB positivity in pulmonary TB patients with specific chest X-ray images but difficulty in expectorating phlegm. In addition, in the future study it can be considered to analyze the comparison of PGE_2_ in TB patients, smokers patients, smokers with tuberculosis, etc.

Nevertheless, this study has several limitations. First, extreme serum PGE_2_ levels were found in some research subjects. This can be caused by various factors that can increase PGE_2_ levels that cannot be controlled. Second, this study only examined PGE_2_ levels in TB patients without comparing them with PGE_2_ levels in healthy persons, so it cannot be used as a predictor factor for the degree of positivity of AFB with sputum.

## 6 Conclusion

The average age of new and relapsed pulmonary TB patients is 43.37 years, mostly male, have a high school education, have a smoking habit, have a low BMI, and have no history of DM. The median serum PGE_2_ level of new and relapsed pulmonary TB patients was 216.95 pg/ml. The majority of new pulmonary TB patients have a high degree of positivity for AFB in sputum, but relapsed pulmonary TB patients have a low degree of positivity for AFB. This study finds a weak negative association between serum PGE_2_ levels and the degree of phlegm AFB positivity but not statistically significant.

## Please state any sources of funding for your research

None.

## Ethical approval

We have conducted an ethical approval base on Declaration of Helsinki at Ethical Committee in Dr. Soetomo General Academic Hospital, Surabaya, Indonesia.

## Consent

Written informed consent was obtained from the patient.

## Author contribution

All authors contributed toward data analysis, drafting and revising the paper, gave final approval of the version to be published and agree to be accountable for all aspects of the work.

## Registration of research studies

1. Name of the registry: Health Research Ethics Coommitee in the Dr. Soetomo General Academic Hospital, Surabaya, Indonesia.
2. Unique Identifying number or registration ID: 1355/KEKP/VII/2019.
3. Hyperlink to your specific registration (must be publicly accessible and will be checked):-.

## Guarantor

Resti Yudhawati.

## Funding

Faculty of Medicine, Universitas Airlangga, Surabaya, Indonesia.

## Provenance and peer review

Not commissioned, externally peer-reviewed.

## Declaration of competing interest

The authors declare that they have no conflict of interest.

## References

[1] (2020). WHO Guidelines Approved by the Guidelines Review Committee. WHO Consolidated Guidelines on Tuberculosis: Module 4: Treatment - Drug-Resistant Tuberculosis Treatment.

[2] Harding E. (2020). WHO global progress report on tuberculosis elimination. The Lancet Respiratory medicine.

[3] Erawati M., Andriany M. (2020). The prevalence and demographic risk factors for latent tuberculosis infection (LTBI) among healthcare workers in semarang, Indonesia. J. Multidiscip. Healthc..

[4] Amaral E.P., Lasunskaia E.B., D'Império-Lima M.R. (2016). Innate immunity in tuberculosis: how the sensing of mycobacteria and tissue damage modulates macrophage death. Microb. Infect..

[5] Mirsaeidi M., Sadikot R.T. (2018). Patients at high risk of tuberculosis recurrence. International journal of mycobacteriology.

[6] Lee J., Hartman M., Kornfeld H. (2009). Macrophage apoptosis in tuberculosis. Yonsei Med. J..

[7] Dietzold J., Gopalakrishnan A., Salgame P. (2015). Duality of lipid mediators in host response against Mycobacterium tuberculosis: good cop, bad cop. F1000prime reports.

[8] Behar S.M., Martin C.J., Booty M.G., Nishimura T., Zhao X., Gan H.X. (2011). Apoptosis is an innate defense function of macrophages against. Mycobacterium tuberculosis. Mucosal immunology.

[9] Ambreen A., Jamil M., Rahman M.A.U., Mustafa T. (2019). Viable Mycobacterium tuberculosis in sputum after pulmonary tuberculosis cure. BMC Infect. Dis..

[10] Lam A., Prabhu R., Gross C.M., Riesenberg L.A., Singh V., Aggarwal S. (2017). Role of apoptosis and autophagy in tuberculosis. Am. J. Physiol. Lung Cell Mol. Physiol..

[11] Kaur H., Chand N., Malhotra B., Singh S.P., Verma V., Thakur S. (2007). Sputum grading as predictor of treatment outcome in pulmonary tuberculosis. Chest.

[12] Hernández-Garduño E., Cook V., Kunimoto D., Elwood R.K., Black W.A., FitzGerald J.M. (2004). Transmission of tuberculosis from smear negative patients: a molecular epidemiology study. Thorax.

[13] Ravimohan S., Kornfeld H., Weissman D., Bisson G.P. (2018). Tuberculosis and lung damage: from epidemiology to pathophysiology. Eur. Respir. Rev. : an official journal of the European Respiratory Society.

[14] Mertaniasih N.M., Kusumaningrum D., Koendhori E.B., Kusmiati T., Dewi D.N. (2017). Nontuberculous mycobacterial species and Mycobacterium tuberculosis complex coinfection in patients with pulmonary tuberculosis in Dr. Soetomo Hospital, Surabaya, Indonesia. International journal of mycobacteriology.

[15] Agha R., Abdall-Razak A., Crossley E., Dowlut N., Iosifidis C., Mathew G. (2019). STROCSS 2019 Guideline: Strengthening the reporting of cohort studies in surgery. Int. J. Surg..

[16] Kumar N.P., Moideen K., Nancy A., Viswanathan V., Shruthi B.S., Shanmugam S. (2019). Plasma eicosanoid levels in tuberculosis and tuberculosis-diabetes Co-morbidity are associated with lung pathology and bacterial burden. Frontiers in cellular and infection microbiology.

[17] Murphy M.E., Phillips P.P.J., Mendel C.M., Bongard E., Bateson A.L.C., Hunt R. (2017). Spot sputum samples are at least as good as early morning samples for identifying Mycobacterium tuberculosis. BMC Med..

[18] Ren S., Li W., Wang L., Shi Y., Cai M., Hao L. (2020). Numerical analysis of airway mucus clearance effectiveness using assisted coughing techniques. Sci. Rep..

[19] Christopher P.M., Widysanto A. (2019). GeneXpert Mycobacterium tuberculosis/rifampicin assay for molecular epidemiology of rifampicin-Resistant Mycobacterium tuberculosis in an Urban Setting of Banten province, Indonesia. International journal of mycobacteriology.

[20] Aziz M.A., Wright A. (2005). The world health organization/international union against tuberculosis and lung disease global project on surveillance for anti-tuberculosis drug resistance: a model for other infectious diseases. Clin. Infect. Dis. : an official publication of the Infectious Diseases Society of America.

[21] Ricciotti E., FitzGerald G.A. (2011). Prostaglandins and inflammation. Arterioscler. Thromb. Vasc. Biol..

[22] Poole E.M., Hsu L., Xiao L., Kulmacz R.J., Carlson C.S., Rabinovitch P.S. (2010).

[23] Schoenberger S.D., Kim S.J., Sheng J., Rezaei K.A., Lalezary M., Cherney E. (2012). Increased prostaglandin E2 (PGE2) levels in proliferative diabetic retinopathy, and correlation with VEGF and inflammatory cytokines. Investig. Ophthalmol. Vis. Sci..

[24] Lo C.J. (2005). Upregulation of cyclooxygenase-II gene and PGE2 production of peritoneal macrophages in diabetic rats. J. Surg. Res..

[25] Amadio P., Baldassarre D., Tarantino E., Zacchi E., Gianellini S., Squellerio I. (2015). Production of prostaglandin E2 induced by cigarette smoke modulates tissue factor expression and activity in endothelial cells. Faseb. J. : official publication of the Federation of American Societies for Experimental Biology.

[26] Chen Y.-J., Lee S.-S., Huang F.-M., Chang Y.-C. (2015). Effects of nicotine on differentiation, prostaglandin E2, and nitric oxide production in cementoblasts. J. Dent. Sci..

